# Eye alignment changes caused by sustained GDNF treatment of an extraocular muscle in infant non-human primates

**DOI:** 10.1038/s41598-020-68743-3

**Published:** 2020-07-17

**Authors:** Jérome Fleuriet, Christy L. Willoughby, Rachel B. Kueppers, Michael J. Mustari, Linda K. McLoon

**Affiliations:** 1 Washington National Primate Research Cente, University of Washington, Seattle, WA USA; 2 Department of Ophthalmology, University of Washington, Seattle, WA USA; 3 Department of Ophthalmology and Visual Neurosciences, University of Minnesota, Minneapolis, MN USA; 4 Graduate Program in Neuroscience, University of Minnesota, Minneapolis, MN USA; 5 Department of Ophthalmology and Visual Neurosciences, University of Minnesota, Room 374 Lions Research Building, 2001 6th Street SE, Minneapolis, MN 55455 USA

**Keywords:** Cell biology, Neuroscience

## Abstract

The ability of sustained treatment of a single extraocular muscle with glial cell line-derived neurotrophic factor (GDNF) to produce a strabismus in infant non-human primates was tested. Six infant non-human primates received a pellet containing GDNF, releasing 2 µg/day for 90 days, on one medial rectus muscle. Eye alignment was assessed up to 6 months. Five of the six animals showed a slow decrease in eye misalignment from the significant exotropia present at birth, ending with approximately 10° of exotropia. Controls became orthotropic. Misalignment averaged 8° three months after treatment ended. After sustained GDNF treatment, few changes were seen in mean myofiber cross-sectional areas compared to age-matched naïve controls. Neuromuscular junction number was unaltered in the medial rectus muscles, but were significantly reduced in the untreated lateral recti. Neuromuscular junctions on slow fibers became multiply innervated after this sustained GDNF treatment. Pitx2-positive cells significantly decreased in treated and contralateral medial rectus muscles. Our study suggests that balanced GDNF signaling plays a role in normal development and maintenance of orthotropia. Sustained GDNF treatment of one medial rectus muscle resulted in a measurable misalignment largely maintained 3 months after treatment ended. Structural changes suggest mechanisms for producing an imbalance in muscle function.

## Introduction

Childhood onset strabismus is a common disorder of eye alignment, and despite intensive study, the cause is unclear in the absence of a known gene defect or injury. Recent gene array and quantitative PCR analyses showed that extraocular muscles obtained from adult subjects with strabismus undergoing normal resection surgery in order to improve eye alignment had significant alterations in gene expression levels and protein composition. These included decreased levels of glial cell line-derived neurotrophic factor (GDNF) compared to levels in age-matched control extraocular muscles^1,2^. This was interesting in light of previous studies which demonstrated that orbital injection of GDNF in juvenile chicks or sustained GDNF treatment of a single extraocular muscle in adult rabbits resulted in altered muscle force generation^3–5^. These studies suggest that modulating the levels of GDNF in selected extraocular muscles may improve eye alignment in individuals with strabismus, and conversely that modulating GDNF levels during early life has the potential to produce a strabismus. This study was designed to test this hypothesis.

A number of well-characterized neurotrophic factors have been demonstrated to modulate extraocular muscle force and myofiber size in rabbits and in chicks^4–9^. These studies formed the basis of a series of experiments examining the ability of neurotrophic factors to produce a strabismus in infant non-human primates or treat strabismus in adult monkeys. Unilateral implantation of insulin-like growth factor-1 (IGF-1) in adult monkeys was sufficient to reduce eye misalignment, thus showing its potential as a treatment strategy for strabismus. In infant monkeys, the main hypothesis being tested was that the normal process of developing orthotropia, which depends on early visual and oculomotor experience and sensitivity, could be modulated through trophic signaling. Of the neurotrophic factors studied thus far, unilateral treatment with insulin-like growth factor-1 (IGF-1) was able to produce a significant strabismus in infant monkeys^10^. In contrast, no strabismus developed after either bilateral medial rectus treatment with IGF-1^11^ or unilateral lateral rectus treatment with brain derived neurotrophic factor (BDNF)^12^.

Based on the demonstration that GDNF levels were altered in the muscles from surgical sample from individuals with strabismus, as shown by using both DNA microarray and RNA PCR analyses^1,2^, sustained treatment of rabbits with GDNF was performed and resulted in muscles that generated decreased force compared to age-matched controls^5^. Based on these results, in the present study we tested the effect of unilateral sustained delivery of GDNF to one medial rectus muscle in six infant monkeys. We hypothesized that manipulation of the levels of GDNF would affect eye alignment, and that these changes would be gradual in onset. GDNF has been localized to the neuromuscular junction^13^, where it plays a role in altering acetylcholine receptor density and the patterning of innervation in development and during regeneration^14–16^. Thus, neuromuscular junction density and pattern of innervation were examined in all the horizontal rectus muscles. The effects of sustained GDNF on the medial rectus muscles suggested that this neurotrophic factor plays a significant role in myofiber remodeling in these muscles. Consequently, the number of Pitx2 myogenic precursor cells was assessed in all the horizontal rectus muscles.

## Results

### Measurements of eye alignment over time

**Figure 1:**
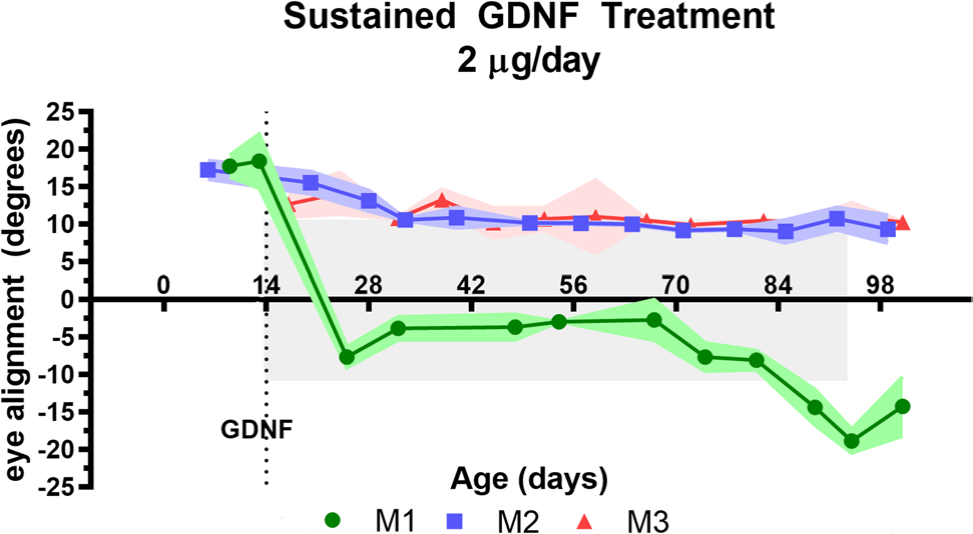
Measurement of eye alignment in degrees every week over the course of 3 months of sustained GDNF treatment for each of the 3 infant monkeys treated. The colored shaded areas represent ± one standard deviation. The gray shaded area represents the duration of GDNF treatment. M1, M2, and M3 refer to each of the 3 three month treated infant monkeys. All the infants in this group were males.

**Figure 2:**
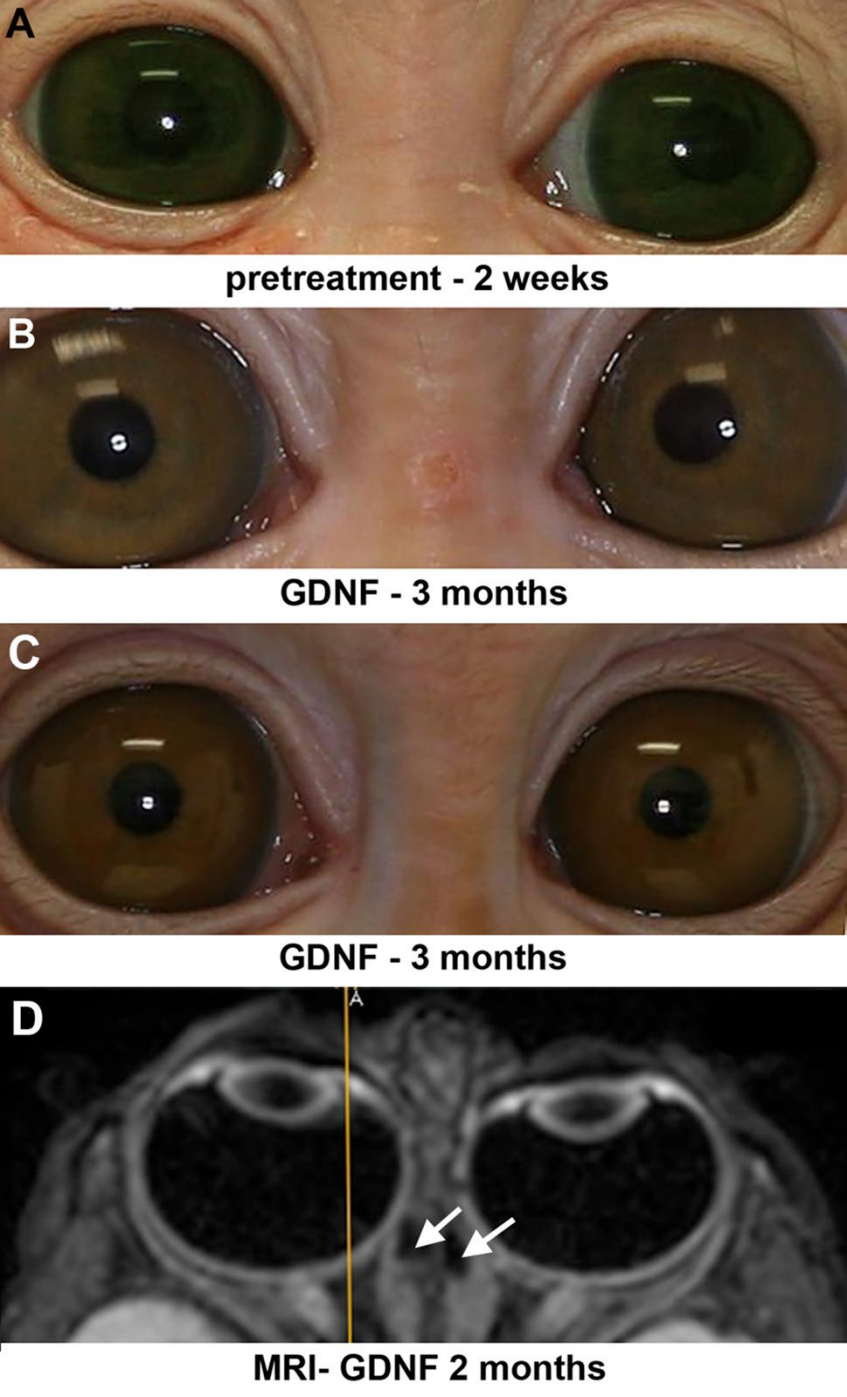
Photomicrographs of monkey 1 prior to treatment at 2 weeks of age (**A**) and at the end of 3 months of GDNF treatment for the one monkey that developed esotropia (**B**), and for one example of one of the monkeys that developed exotropia (M2) (**C**). (**D**) Magnetic resonance imaging (MRI) image of monkey 3 two months after implantation of a GDNF-releasing pellet on the right medial rectus muscle and a placebo control pellet on the left medial rectus muscle. The yellow line was generated by the MRI image acquisition system to indicate orientation. A indicates anterior. All monkeys in this group were male. Arrows indicate pellets on the medial rectus muscles.

**Figure 3:**
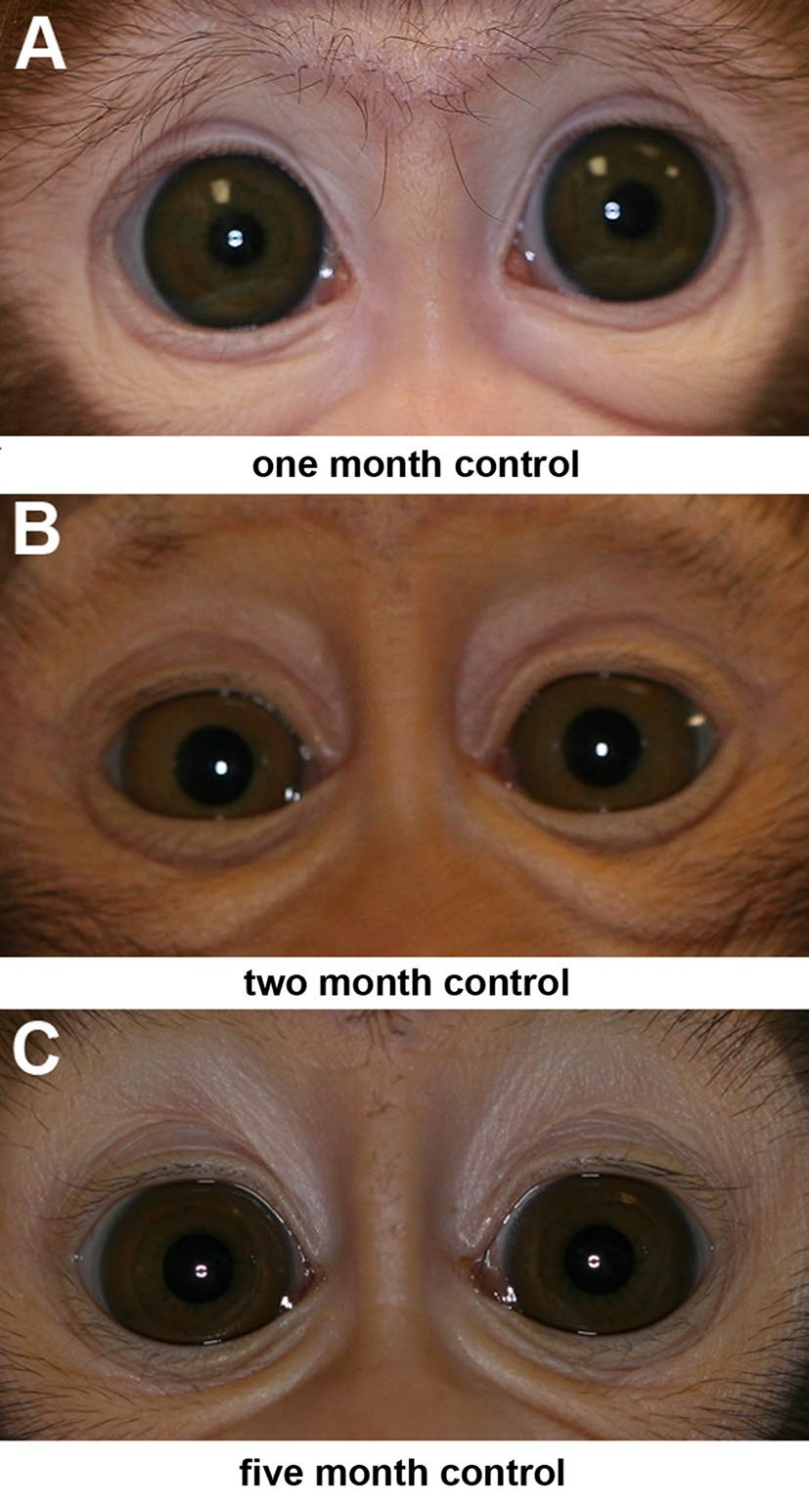
Corneal light reflections from naïve control infant monkeys at (**A**) 1 month (female), (**B**) 2 months (male), and (**C**) 5 months of age (female).

For the 3 naïve infant monkeys, at one month the eye misalignment was significant and mirrored that of the 6 infants in the current study as well as 6 other infant monkeys from previous studies where eye alignment was tracked over time^10,12^. The naïve monkeys at one month had an exotropia of 12.6°, similar to the misalignments in infant monkeys between birth and one month (Figs. 1, 2, 3). Over time, the animals became orthotropic. By 2 and 5 months, the infant-associated exotropia seen in all the infant non-human primates was reduced to 1.6° and 3.5°, angles below what is defined as strabismus (Fig. 3), adding support to the data showing maintained or induced eye misalignment in the GDNF-treated infant monkeys.

All three infant monkeys that were treated for 3-months duration only were exotropic in the first or second week of life, with two infant monkeys at approximately 17.5° of exotropia and the third at 12.5° of exotropia (Fig. 1). One major pattern of eye alignment change emerged over time. Two of the 3-month treated infants progressed toward approximately 10° of exotropia within the first month, where they remained for the next 2 months during sustained GDNF treatment. One of the infants was highly exotropic, progressed toward normal eye alignment, ending the first month at approximately 3° of esotropia. However, at the end of 3 months, eye alignment became progressively more esotropic and ended the third month the most misaligned of the three treated infants, with a misalignment of 19° of esotropia (Figs. 1, 2). Pellet placement was verified using magnetic resonance imaging (MRI), and all pellets were located and found to be in place on the medial rectus muscles (Fig. 2D). The mean absolute eye misalignment of these three infant monkeys at the end of three months of GDNF treatment was 12.8° ± 3.1°, two exotropic and one esotropic (Fig. 1).

**Figure 4:**
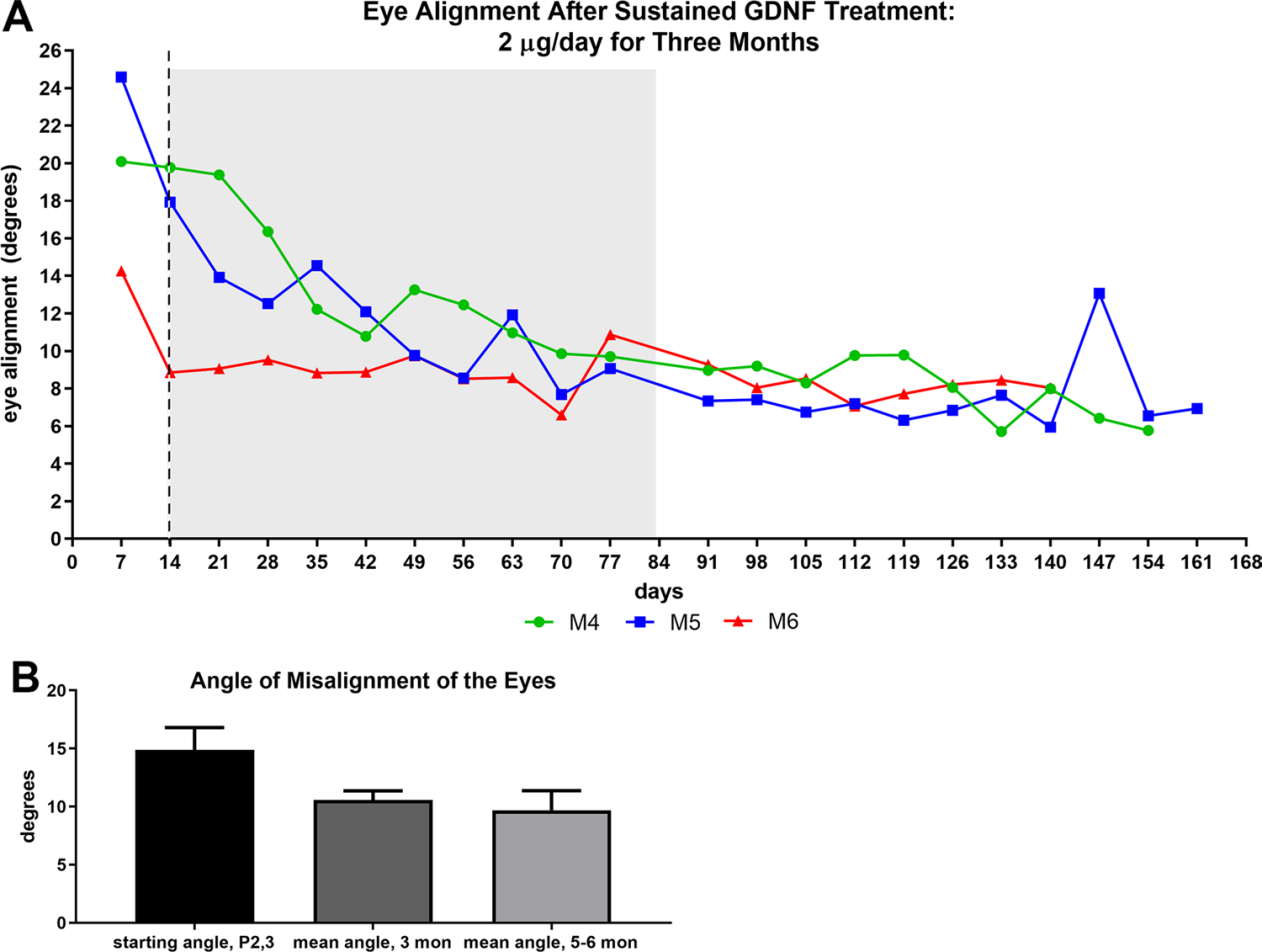
(**A**) Measurement of eye alignment in degrees every week over the course of the 3 months of sustained GDNF treatment (represented by gray rectangle) and 3 months of continued observation (release period ended) in three additional infant monkeys (two male, 1 female). (**B**) Mean of eye alignment, in degrees, from the first measurement, at 3 months, and the averaged final measurements at 5–6 months. There were two males and one female (M5) in this group.

A second group of three infant monkeys was treated with sustained release of GDNF delivered to the medial rectus muscle for 3 months, and then were followed for an additional 3 months after GNDF release ended (Fig. 4). Photographs to determine eye alignment were taken every week during treatment and 3 months after, totaling 6 months (Fig. 4). Eye alignment was exotropic (24.6°, 20.1°, and 14.3°) at the beginning of treatment, and misalignment was maintained between 8.2° and 9.7° exotropia at the end of the 3-month treatment with sustained release GDNF (gray box, Fig. 4A). The mean angle of misalignment of all 6 monkeys after 3 months was 10.58° ± 0.8°. At the 6 month time point, 3 months post -GDNF treatment, a micro-strabismus was maintained in all 3 monkeys, with a mean of 9.67° ± 1.68° when the final 3 mean angles for each monkey were averaged (Fig. 4A, B). In normal monkeys, by 3 months of age the eyes were all orthotropic (Fig. 3)^17^.

### Myofiber cross-sectional area measurements

**Figure 5:**
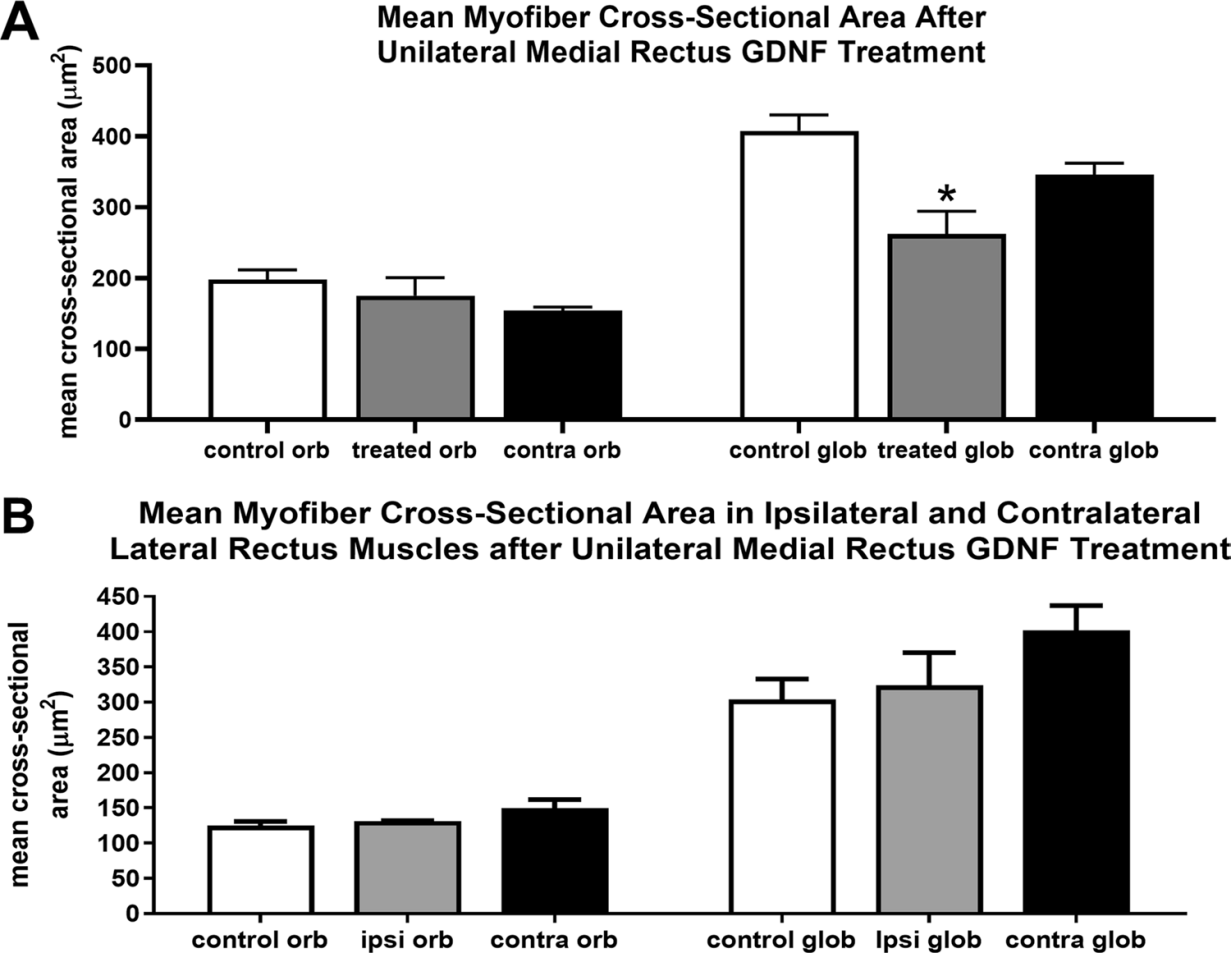
(**A**) Mean myofiber cross-sectional areas in the orbital and global layers in naïve control medial rectus muscles (white bars), treated medial rectus muscles (gray bars), and the medial rectus muscles contralateral to the muscle treated (contra) (black bars). (**B**) Mean myofiber cross-sectional areas in the orbital and global layers in naïve control lateral rectus muscles (white bars), the antagonist lateral rectus muscles on the treated side (ipsi) (gray bars), and the lateral rectus muscles on the contralateral side (contra) (black bars). Data are reported as mean ± standard error of the mean. *Indicates significant difference from naïve control muscles in the same muscle layer.

Mean myofiber cross-sectional areas were calculated in all four horizontal rectus muscles (Fig. 5). In all the orbital layers, no significant changes were seen. In the global layers, the mean myofiber cross-sectional areas were significantly smaller than the age matched control. The global layer mean myofiber cross-sectional areas were decreased by 35.7% (p = 0.009) (Fig. 5A). The mean cross-sectional myofiber areas of medial rectus muscles on the contralateral side were not significantly different from the control muscles. There were no significant differences between the mean myofiber cross-sectional areas of the control medial rectus muscles and the medial rectus muscles on the side contralateral to the treatment. Neither were there any significant differences in the mean myofiber cross-sectional areas between any of the lateral rectus muscle pairs, in either the orbital or global layers (Fig. 5B).

### Neuromuscular junctions

**Figure 6:**
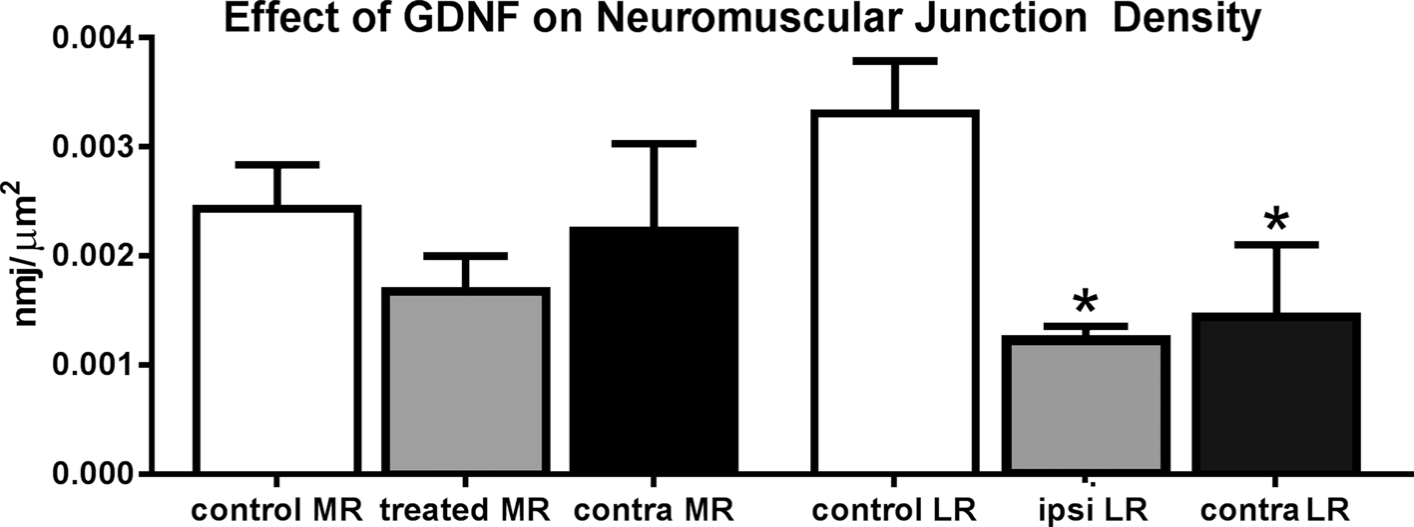
Density of neuromuscular junctions (nmj) in naïve control medial (MR) and lateral (LR) rectus muscles (white bars), treated MR muscles (gray bar), MR muscles contralateral to the treated MR muscles (black bar), the LR on the same side as the treated MR muscles (ipsi LR) (gray bar), and the lateral rectus on the contralateral side (contra LR) (black bar). Data are reported as mean ± standard error of the mean. *Indicates significantly different from naïve control muscles.

The GDNF receptor is found at the neuromuscular junction^13^ and plays a role in distribution of acetylcholine receptors and the patterning of their innervation^13,14^. We examined neuromuscular junction density based on number of neuromuscular junctions per mm^2^ tissue as a basic measure of changes to their overall density (Fig. 6). There were no significant differences in overall neuromuscular junction density in the GDNF-treated medial rectus muscles or the untreated medial rectus muscles on the contralateral side. This was the case, despite 36.1% fewer neuromuscular junctions per mm^2^ tissue in the treated medial rectus muscle compared to naïve control muscles. However, both the antagonist lateral rectus muscle and the contralateral lateral rectus muscle had significantly fewer neuromuscular junctions per mm^2^ tissue, decreases of 89.8% and 77.5% respectively, than the naïve age-matched control lateral rectus muscle (Fig. 6).

### Innervation

**Figure 7:**
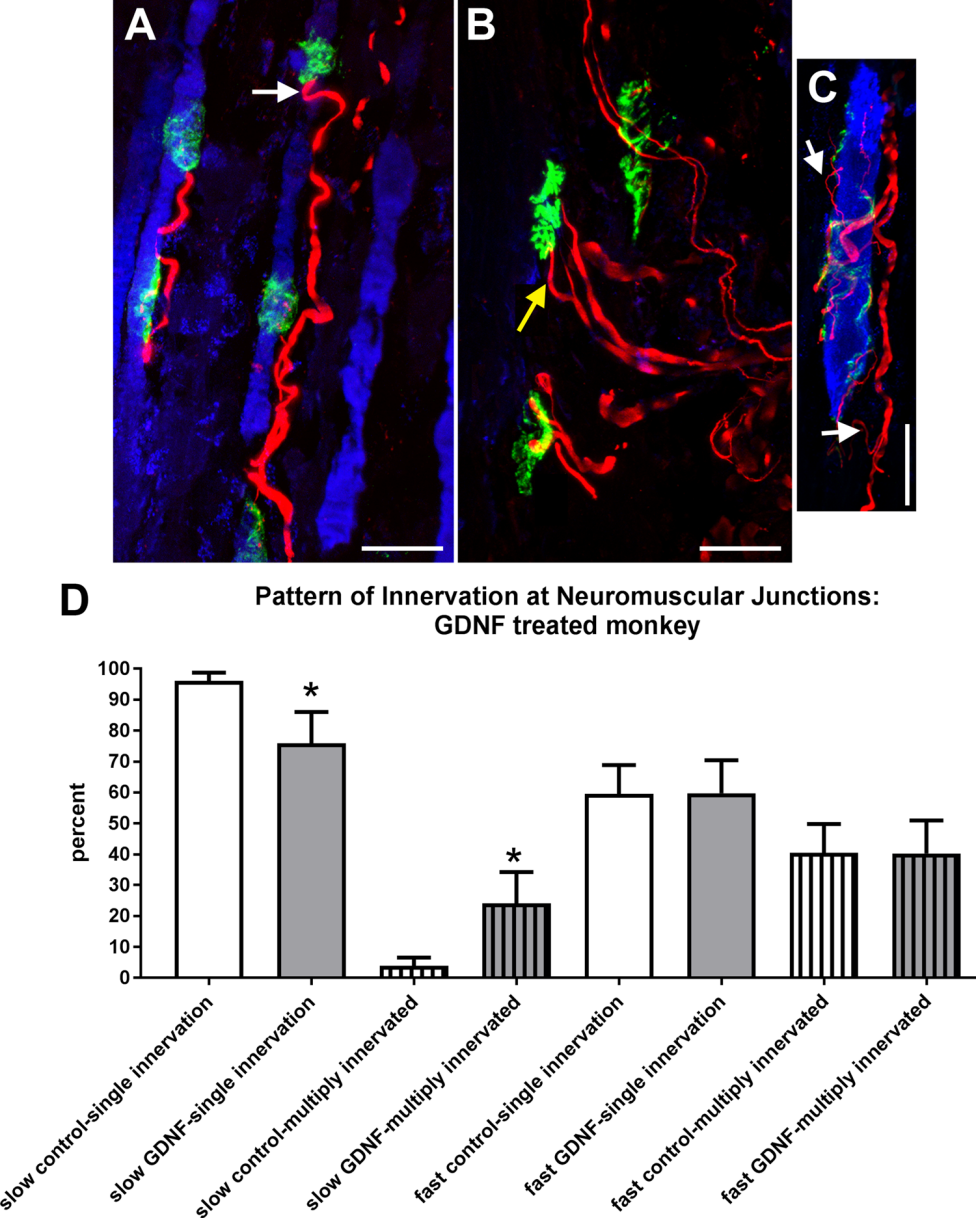
(**A**) Confocal image of a longitudinal section through the medial rectus of a naïve control 3-month old monkey. Arrow indicates an axon. Magnification bar is 30 µm. (**B**) Confocal image of a longitudinal section through the medial rectus muscle of an infant monkey after 3 months of sustained GDNF treatment showing multiple axons running to three different *en plaque* neuromuscular junctions on slow myosin heavy chain isoform negative fibers. Yellow arrow indicates two axons connecting to a single neuromuscular junction, which were traced using confocal microscopy. (**C**) Confocal image of a longitudinal section through the medial rectus muscle of an infant monkey after 3 months of sustained GDNF treatment showing multiple axons connecting to a single neuromuscular junction in a neuromuscular junction on a slow-positive myofiber. White arrows point to typical thin “extra” axons projecting to the neuromuscular junction. Blue: slow myosin heavy chain isoform. Green: α-bungarotoxin. Red: neurofilament protein. Magnification bar for all images is 30 µm. (**D**) Analysis of percent of neuromuscular junctions with either single (solid bars) or multiple-innervation (striped bars) in naïve control medial rectus muscles (white bars) and GDNF-treated medial rectus muscles (gray bars) for both slow MyHC-positive and -negative myofibers. Data are reported as mean ± standard error of the mean. *Indicates significant difference from control muscles.

We assessed the pattern of innervation at neuromuscular junctions for both slow MyHC-positive and negative myofibers in control and GDNF-treated medial rectus muscles using both regular fluorescence microscopy and confocal microscopy (Fig. 7, Supplemental video 1). There was a significant fivefold increase in the percent of neuromuscular junctions with more than one axon in the GDNF-treated muscles compared to the naïve control muscles, from 4.2% to 21.3% of neuromuscular junctions, which were multiply innervated. There were no significant changes to the pattern of innervation of the fast MyHC-positive myofibers after GDNF-treatment compared to the naïve control muscles.

### Myogenic precursor cells

**Figure 8:**
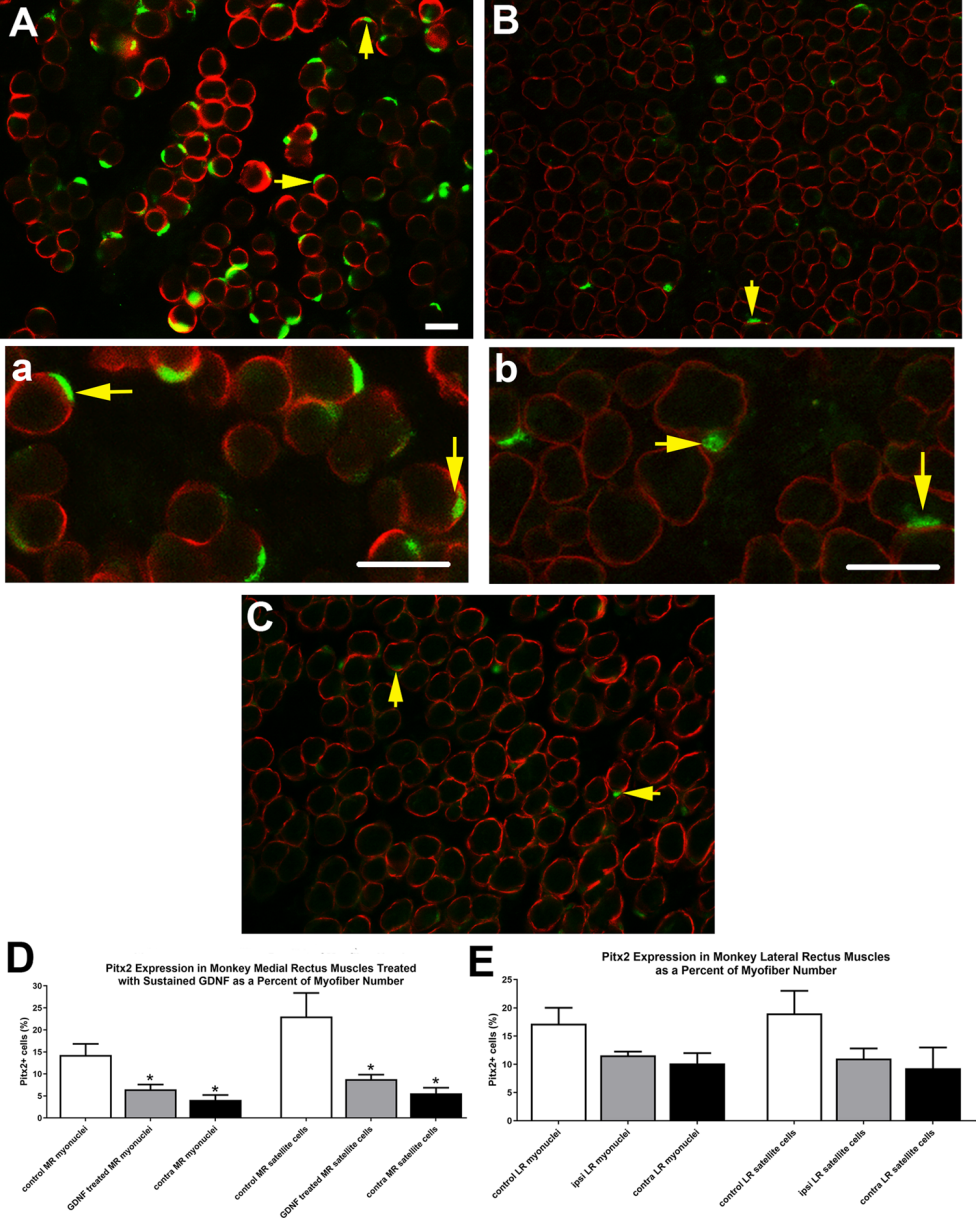
Photomicrographs of (**A**, **a**) a naïve control medial rectus muscle, (**B**, **b)** a GDNF-treated medial rectus muscle, and (**C)** a medial rectus muscle contralateral to the treated muscle immunostained for the expression of Pitx2 (green) and dystrophin (red). Vertical arrows indicate examples of Pitx2-positive myonuclei and horizontal arrows indicate Pitx2-positive cells outside of the sarcolemma. Magnification bar is 30 µm. (**D**) Quantification of the percent of Pitx2-positive myonuclei and Pitx2-positive cells outside of the sarcolemma relative to fiber number for the naïve control (white bars), GDNF-treated (gray bars), and contralateral medial rectus muscles (black bars). (**E**) Quantification of the percent of Pitx2-positive myonuclei and Pitx2-positive cells outside of the sarcolemma relative to fiber number for the lateral rectus muscles from naïve control orbits (white bars), on the same globe as the treated medial rectus muscle (ipsi) (gray bars), and the contralateral lateral rectus muscles (contra) (black bars). Data are reported as mean ± standard error of the mean. *Indicates significant difference from the naïve control levels.

It has previously been shown that the extraocular muscles remodel throughout life^18–20^. We have postulated that this is due to elevated levels of myogenic precursor cells^21^, in particular a muscle stem cell that expresses Pitx2^22^. The effect of sustained GDNF treatment on Pitx2-positive cells was analyzed. After 3 months of sustained GDNF treatment there was a significant 54.5% decrease in the number of Pitx2-positive myonuclei and a 61.9% decrease in the number of Pitx2-positive cells outside the sarcolemma in the treated medial rectus muscles (Fig. 8A, B, D). Interestingly, in the medial rectus muscle on the side contralateral to the GDNF treatment, there was a significant decrease in these Pitx2-positive populations of cells, with a 71.3% decrease in the number of myonuclei as a percent of myofiber number compared to the naïve control levels, and a similarly significant 75.8% decrease in Pitx2-positve cells outside the sarcolemma (Fig. 8C, D). These decreases in the Pitx2 population correlated with the decreased mean myofiber cross-sectional areas seen in the global layers of the medial rectus muscles that had been treated for three months. The Pitx2 populations were assessed in the lateral rectus muscles in the orbits ipsilateral and contralateral to the treated medial rectus muscle and compared to age-matched naïve control lateral rectus muscles (Fig. 8E). Both the ipsilateral and contralateral lateral rectus muscles had decreases of 26.5% and 40.6% in Pitx2-positive myonuclei and decreases of 40.4% and 50.1% in the Pitx2-positive cells outside the sarcolemma, respectively, from the percentages seen in the age-matched naïve control lateral rectus muscles. However, none of these analyses of the lateral rectus muscles showed a statistically significant difference.

## Discussion

Sustained treatment with GDNF to one medial rectus muscle in infant monkeys resulted in the development of a significant strabismus, which at 3 months averaged 10.58° of misalignment in the 6 treated infant monkeys. Even 3 months after GDNF treatment ended, the monkeys retained a microstrabismus with misalignments averaging 6.9 degrees of exotropia. Our previous study using sustained release of IGF-1 resulted in a larger average misalignment of 13.03 degrees at the end of 3 months^17^. A possible bias known as angle kappa could overestimate exotropia in these monkeys. Angle kappa has been defined as the angle between the line of sight and the anatomic orientation of the eye or corneal/pupillary axis^23^. In human infants, it was shown that this angle decreases during development^24^, suggesting a risk for overestimating exotropia in young human infants. The measurements of eye alignment of three naïve control infants showed a 12.6° exotropia for the 1-month-old infant and a near alignment for the 2 and 5-month-old infants (1.5° and 3.5° respectively). Even though the exotropia found is in agreement with a study showing that normal non-human primate infants started out exotropic at birth and slowly progressed toward alignment over this 3-month period after birth^25^, this exotropia might be due to a larger kappa angle for young nonhuman primate infants as reported in human infants. However, a reliable average estimation of angle kappa for nonhuman primate infants would require a large cohort of controls, as was done in the original study of 323 human infants^24^. The use of angle kappa also would require that perfect eye coordination and alignment is effective right after birth. There are no data that support this view, particularly when one considers the time course of development of binocularity in infant primates.

Many research studies in nonhuman primates suggest that normal visual experience during the first 6 weeks of life is necessary for development of normal binocular visual acuity and eye alignment over time^26–34^. Further support for this view is based on the three naïve control infants included in the current study, which showed almost perfect eye alignment at 2 and 5-months of age. We argue that the changes in eye alignment after 2 months of development were a direct effect of the sustained GDNF treatment. This is supported by the lack of development of strabismus in infant monkeys treated with BDNF^12^ or when IGF-1 pellets were implanted in infant monkeys bilaterally^11^. If pellets alone caused perturbations in alignment, then contralateral implantation of the placebo pellet would negate the effects of the first pellet, which was not seen. Similarly, in rabbit studies, injections of hepatocyte growth factor (HGF) alone did not cause changes to the muscle physiology or myofiber morphometry, while injections with IGF-1 produced significant changes in muscle function and protein expression, and injection with HGF followed by IGF-1 produced even greater significant changes to myofiber force generation and myofiber mean cross-sectional areas^35^. In our studies in rabbit, we included cases with just saline injections compared to neurotrophic factor injections and found no functional changes in these sham-treated muscles^36^.

Several interesting observations come from these data. First, similar to the infant monkeys treated unilaterally with IGF-1, one major pattern of change in eye alignment was seen over time after these sustained neurotrophic factor treatments. The common pattern of change in eye alignment over time after sustained IGF-1 or GDNF treatment was a slight decrease in exotropia in the first two weeks followed by maintenance of misalignment for the duration of treatment. A second pattern was seen in 2 of the 9 infants treated with either IGF-1 or GDNF studied thus far, where there was a relatively rapid decrease in their exotropia within the first 2–4 weeks, followed by an increase in strabismus angle between 2 and 3 months. In the current study, one infant monkey (Figs. 1, 2) was initially exotropic, yet ended up significantly esotropic by the end of 3 months of treatment. It is important to note that similar differences in the ultimate type of strabismus, exotropia or esotropia, is seen after all the current methods used for producing strabismus in infant non-human primates: alternating monocular occlusion, prism rearing, and muscle surgery^37–44^. Similarly, instability in ocular alignment is relatively common in children with strabismus^45,46^, so it was expected that we would see similar instability in the weekly eye alignment in some of the infant monkeys in our study. The mechanisms behind such differential outcomes are currently unclear. One such study examined the change in eye alignment and saccade parameters after strabismus surgery in prism-reared adult strabismic monkeys, both with exotropia. The two monkeys showed distinctly different patterns of saccade gain in response to the resection/recession surgery performed in one orbit^47^, despite similar causes and treatment of their eye misalignment. Recent work from the Das laboratory provides strong evidence that these differences after surgery are under motor neuron control, and the net result was to decrease the surgical correction^48,49^. How this differential motor drive is controlled at the molecular level is a subject that requires significant future study.

The examination of GDNF was based on published DNA microarray and quantitative PCR analyses showing the GDNF was differentially expressed in adult subject extraocular muscles from subjects with strabismus^1,2^. The development of strabismus after 3 months of GDNF treatment in infant monkeys supports the potential role of GDNF in the onset and/or maintenance of strabismus. Combined, these data suggest that endogenous and potentially dysregulated GDNF expression may be involved in the molecular control in the development and/or maintenance of eye misalignment in early life. Muscle force was significantly reduced after one month of sustained GDNF treatment of adult rabbit extraocular muscles^5^. Mean twitch contraction times were significantly increased compared to naïve control muscles in the treated muscles, which would result in a more sustained, albeit weaker, tension in the treated muscles^5^. In young chick extraocular muscles, GDNF injection decreased contraction duration^11^. These data suggest that the effect of GDNF may change over the period of muscle and oculomotor system maturation and the critical period for the development of binocularity^26–34^. Data suggest that the visual system may be even more sensitive to binocular inhibition when strabismus develops during the specific time when stereopsis normally merges^50^. Further studies are needed to determine the effects of maturation on treatment effects.

GDNF is retrogradely transported to motor neurons, where it serves as a trophic factor during development and after injury^51,52^. The current data from the treated infant monkeys, as well as previous studies in developing chick extraocular muscle^3^, suggest that the GDNF also has actions directly at the muscle level. While GDNF is known to be expressed in skeletal muscle^53^, there are few studies that have examined the role GDNF might play in specifically altering the structure and function of skeletal muscle. In the current study, a continuous exposure of a single medial rectus muscle to GDNF for three months resulted in decreased mean myofiber cross-sectional area in the global layer of the treated medial rectus muscle when compared to naïve age-matched control medial rectus muscles from infant monkeys. This suggests that mean cross-sectional area is not the most significant variable in the effects of GDNF on extraocular muscle.

Two other neurotrophic factors implicated in the gene expression studies were IGF-1 and ciliary neurotrophic factor (CNTF). IGF has been demonstrated to have modulatory effects on extraocular muscle size and force generation capacity in chick, rabbits, and non-human primates^2–7,10,54^. Decreased levels of CNTF, GDNF, as well as altered levels of IGF binding proteins, which are inhibitory in function, shown in the microarray studies^1,2^ suggest that increasing the levels of a single neurotrophic factor is likely to be insufficient to generate or treat a large angle strabismus. This is compounded by the fact that GDNF is normally expressed in extraocular muscles both during development^52^ and in aging^55^, and at higher levels than seen in limb skeletal muscle^9^. By providing an excess level of GDNF in the infant medial rectus muscles using our sustained release method, we were able to disturb the normal neurotrophic factor equilibrium sufficiently to perturb the normal process of eye alignment in these infants.

One of the main functions of GDNF in motor systems is to alter nerve growth and neuromuscular junction size and function^15,56^. In developing systems, GDNF specifically increased nerve growth and enhanced synaptic transmission^57^. We found that in the presence of increased GDNF, the main effect on numbers of neuromuscular junctions was an adaptive response on the contralateral side. These types of adaptive changes in the absence of direct neurotrophic factor treatment are described in many studies where unilateral treatment was applied. Our own work showed significant coordinated changes contralateral to unilateral treatment with IGF-1^10^, after resection surgery^58^, and after recession or tenotomy surgery^59^. Similarly after resection surgery in strabismic non-human primates significant coordinated changes in motor drive were seen on the untreated contralateral side^47,48^. These studies suggest significant communication between extraocular muscles and the oculomotor brainstem circuits.

Sustained treatment with GDNF in the infant monkey medial rectus muscles resulted in a significant increase in multiply innervated neuromuscular junctions on slow myofibers specifically. These results are supported by previous studies demonstrating that in GDNF-overexpressing muscles, neuromuscular junctions became “hyperinnervated”^16^ with many of these additional axons thinner than normal^60^, which was also seen in the present study. The polyinnervated neuromuscular junctions in the GDNF over-expressing mouse muscles were electrophysiologically weak^60^. This is interesting in light of the decreased force generation in GDNF-treated rabbit extraocular muscles^5^. Further work is needed to assess functional and protein changes specifically at the neuromuscular junctions after GDNF treatment. Whatever the exact mechanism, the end result was that 3 months of sustained GDNF treatment resulted in a strabismus in these infant monkeys.

Pitx2 myogenic precursor cells are highly expressed in extraocular muscles^22^, and are hypothesized to play an important role in the continuous remodeling that occurs in these muscles throughout life^20,21,61^. Interestingly, GDNF mRNA increased in injured skeletal muscle with increased expression specifically in muscle satellite cells^62^. While GDNF, in the short term, resulted in increased numbers of Pitx2-positive cells in adult rabbit extraocular muscle^5^, in contrast, GDNF treatment over a period of 3 months in extraocular muscles in infant monkeys that were not yet mature caused a significant decrease in Pitx2-positive cells in both the treated and untreated medial rectus muscles. The impact of these changes on overall extraocular muscle function is unclear.

The question of specificity of this GDNF treatment and the subsequent appearance of strabismus is a commonly raised concern. If a neurotrophic factor containing sustained release pellet, with a contralaterally-placed placebo pellet, caused strabismus all by themselves, then regardless of the neurotrophic factor delivered, strabismus would ensue. However, this is not the case. Bilateral IGF-1 treatment of both medial rectus muscles, with placebos on the lateral rectus muscles, did not cause strabismus^11^. In this study compensatory changes in the untreated lateral rectus muscles occurred bilaterally, presumably resulting in the unchanged eye alignment that resulted^11^. Similarly, unilateral treatment with BDNF did not cause strabismus, despite causing a number of changes to muscle morphology^12^. In contrast, after unilateral treatment of one medial rectus muscle with IGF-1 in infant monkeys, we saw significant changes in the treated and untreated contralateral medial rectus muscles, but no significant compensatory alterations in the lateral rectus muscles^10^. These types of compensatory changes in antagonist muscles occurred after a number of perturbations of the normal equilibrium in the extraocular muscles. In unilateral procedures in particular, performing tenotomy on only one muscle produced significant antagonist weakening in cat extraocular muscle^63^. In a recent study from the Das laboratory, changes to oculomotor and abducens motor drive after resection and recession surgery in strabismic monkeys were examined over a 6 month period after surgery of adult strabismic monkeys^47^. Similar disparate changes were seen in the motor drive after surgery, where significant changes were seen only in one motor nucleus^37^, and over a period of 6 months eye alignment reverted from the surgical correction and became more strabismic. While our original hypothesis was that these growth factors would result in “larger or smaller” muscles that would then produce altered force, it appears that the adaptations of the extraocular muscles to these perturbations are more complex than simple alterations in muscle size. Collectively our studies, and those of others, support the view that remodeling and adaptation of these muscles likely play an important role in the development of strabismus in what are apparently “normal” muscles^64^. These studies suggest that in order to prevent or treat strabismus, addressing both extraocular muscle and their innervating motor neurons will be critical^65^.

In summary, this study demonstrates that continuous treatment of single infant monkey medial rectus muscles produces eye misalignment that remains at least as long as 3 months after cessation of treatment. Based on these results, as well as the DNA microarray studies^1,2^, future studies will examine the co-administration of neurotrophic factors to produce eye misalignment in infant non-human primates or improve eye alignment in adult strabismic monkeys.

## Methods

Studies were approved by the animal care and use committees at the University of Washington and the University of Minnesota. All experiments followed the recommendations of the ARVO Guidelines for Use of Animals in Ophthalmic and Vision Research and the NIH Animal Care and Use Guidelines. A total of nine monkeys were included in this study: (1) three normal infants with no treatment who were used to establish a baseline for development of eye alignment; (2) three that were treated for 3 months prior to euthanasia and examination of their extraocular muscles, and (3) three that were used to follow eye alignment changes, and thus were treated for 3 months and followed for an additional three months but on whom no histology was performed.

Pellets containing GDNF with a release profile of 2 µg/day were manufactured for this study by Innovative Research of America (Sarasota, FL). This dosing was based on previous dose–response studies in rabbit examining the effects on muscle force generation of injecting IGF-1 in a series of increasing concentrations^36^. Injected doses of 1 and 5 µg were shown to cause significantly upregulated muscle force generation^36^, with comparable changes in muscle when compared to pellet implantation for the same time period^7^. Saline injections alone caused no significant alteration in generated muscle force^7^. Calculations can be made to estimate the actual daily level of GDNF present in the muscles after pellet implantation. The amount of endogenous GDNF present in extraocular muscle is thought to be about 10–20 ng/g, based on reports demonstrating that levels of GDNF mRNA are about 10–20 fold higher than in limb skeletal muscle^11^, assuming that the level of GDNF mRNA and GDNF protein in muscles increases in a linear fashion^16^. In previous work on developing chick extraocular muscle, a single dose of 5 µg GDNF resulted in retention of about 400 ng/g in the extraocular muscle^11^. This dose was similar to that used for IGF-1, where effective doses after injection of 0.5 and 5 µg IGF-1 into the chick orbit were 40 ng/g and 400 ng/g, respectively. Since in the experiments, 2 µg/day were released over 3 months, it can be estimated that the effective and sustained dose was similar to the injection of 0.5 µg, which resulted in an effective dose of approximately 40 ng/g. Accordingly, we estimate that our treatment with exogenous GDNF increased total GDNF levels by approximately 2—fourfold, from 10–20 ng/g to about 50–60 ng/g, assuming that there was no alteration of endogenous GDNF levels due to the treatment.

Using sterile procedures, infant Macaca nemestrina monkeys, on average 14 days after birth, were anesthetized using sevoflurane inhalation anesthesia, intubated, and maintained on 2.5%-3% sevoflurane, performed by veterinary staff at the Washington National Primate Center. All infants were implanted with the sustained release pellets containing human GDNF (R and D Systems, Minneapolis, MN) with a release profile of 2 µg/day for 90 days, and all infants received a placebo pellet on the contralateral side. Timing is consistent with previous work where strabismus was generated^34^. GDNF is highly conserved, and based on analysis using https://blast.ncbi.nlm.nih/gov/Blast.cgi, there is a significant homology between species. As previously described^10^, under general anesthesia the conjunctiva was opened near the limbus in the area of the medial rectus muscle. After visualization of the muscle, a small muscle hook was placed under the muscle at its insertion, and the GDNF-containing pellet was placed on one medial rectus muscle. These pellets are quite adhesive when wet, and stay in the location where they are placed. The conjunctiva was then sutured closed using ophthalmic 8-0 vicryl suture. MRI was used to verify the retention and position of the pellet at two months post-implantation (Fig. 2)^10^. For the MRI, the monkeys were anesthetized, as described for pellet-implantation surgery, by veterinary staff, and the head was supported with towels during the actual MRI acquisition. Hand-held infant monkeys were photographed in primary position of gaze prior to pellet implantation to verify starting eye alignment and then every week for a duration of 3 months for 3 monkeys (all male), and measured every other week for another 3 months after release ended for a total duration of up to 6 months for the second group of 3 monkeys (2 males, 1 female). On several occasions, an individual monkey was too difficult to handle safely; this animal was returned to its caretaker and retested a week later. As these monkeys were handheld, one monkey became too difficult to handle safely at the end of five months. At the end of this extended time period for photographic visualization to determine eye alignment, these three monkeys were returned to the primate colony at the Washington National Primate Center. There were five males and one female monkey. These photos were used to assess eye alignment by calculating the displacement between the pupil center and the reflection from the flash for each eye. This allowed the determination of the degree of eye alignment based on the Hirschberg Ratio, which for macaques is 14° per millimeter^25^. This is a well-established method for determining eye alignment used in human infants on a regular basis, whose use is supported by the American Academy of Ophthalmology and the American Association for Pediatric Ophthalmology and Strabismus. We averaged the measurements from three to eight photographs for each time point and for each subject. Three naïve control infant monkeys at one month (female), two months (male), and five months (female) of age were photographed, and eye alignment determined in a similar manner.

At the end of 3 months, the first group of monkeys was euthanized with an overdose of barbiturate anesthesia, and all the extraocular muscles were removed and immediately embedded in tragacanth gum and frozen in liquid nitrogen-cooled 2-methylbutane and stored at − 30 °C until sectioned. In addition, three 3-month old infant monkeys were collected from infants that were rejected by their mothers or accidently injured in the Washington National Primate Center and used to produce a baseline for the normal development of eye alignment in infant monkeys. In a cryostat, 10 µm sections were prepared through all the horizontal extraocular muscles. Every 30^th^ section was stained with hematoxylin and eosin for determining mean cross-sectional areas. Using Bioquant Life Science Image Analysis System version 19.5.6 (Bioquant Image Analysis Corporation, Nashville, TN), cross-sectional areas were manually traced for 200 muscles fibers in the orbital layer and 300 muscle fibers in the global layer for each of three sections per muscle per animal. The averages of the 3 treated and 3 naïve control animals were determined and used for statistical analysis.

To visualize neuromuscular junctions, tissue sections were rinsed in phosphate buffered saline (PBS), followed by an incubation with α-bungarotoxin conjugated to Alex Fluor 488 (AF488; 1:500; Invitrogen; Carlsbad, CA; B13422) in PBS, rinsed, and coverslipped. All neuromuscular junctions were counted through three entire sections randomly selected along the length of each muscle avoiding the extreme tendon ends and the middle as identified by embryonic MyHC isoform immunostaining in serially stained sections. The total area of each section as visualized using the imaging system was measured, which determined the number of neuromuscular junctions per mm^2^ of tissue. These measurements included all tissues within any given microscopic field, avoiding epimysium and any areas where histological artifacts might be present. For each muscle, these values were averaged, and these averages were used to determine the means for each set of muscles. To assess whether there were changes to the pattern of innervation at the neuromuscular junction, sections were immunostained for expression of slow myosin, neurofilament, and for the neuromuscular junction. Slides were rinsed in PBS, blocked in 20% goat serum and 0.2% bovine serum albumin (BSA), incubated with a mouse antibody to slow twitch heavy chain isoform (MyHC) (1:200; Hybridoma Bank; A4.951-c) in PBS containing 0.1% Triton x-100 (Ab buffer) for one hour at room temperature, rinsed in PBS, and incubated for 30 min at room temperature in a goat anti-mouse Dylight 405 secondary antibody (1:100; Jackson ImmunoResearch, West Grove, PA; 115–475-146). The sections were rinsed in PBS and blocked with goat anti-mouse Fab fragments (1:100; Jackson ImmunoResearch; 115-166-003) overnight at 4 °C. The following day the slides were rinsed in PBS, blocked with goat serum, and incubated with a mouse antibody to neurofilament protein, anti-smi31 (1:5,000 in Ab buffer; BioLegend, San Diego, CA; 801601) for 90 min at room temperature. The slides were rinsed in PBS and incubated in goat anti-mouse Cy3 (1:500; Jackson ImmunoResearch; 115-165-146) for one hour at room temperature. After another PBS series of rinses, the sections were incubated in α-bungarotoxin conjugated to AF488 (Invitrogen). After a PBS rinse, the slides were cover-slipped and examined using scanning laser confocal microscopy at X40, and images were captured at X60 using oil immersion (FV1000; Olympus Corp., Tokyo, Japan). We sequentially captured images in the same focal plane using different filters for the three fluorochromes used. For each neuromuscular junction, the Z-stacks were collapsed. Neuromuscular junctions were chosen in the stained sections at random and analyzed for number of axons per neuromuscular junction examined for both slow and fast myofibers. An average of 28 neuromuscular junctions were assessed per GDNF-treated medial rectus muscle for each of the three treated monkeys and an average of 63 neuromuscular junctions were assessed per naïve control medial rectus muscle.

Pitx2 and dystrophin were visualized on tissue sections immunohistochemically, selecting regions spaced evenly along the muscle length where a number of muscle fibers were in cross-section. Each muscle was washed in PBS, blocked in 20% normal goat serum, followed by incubation in a mouse antibody against Pitx2 (1:400, abcam, Cambridge, MA; ab55599) for 1 h at room temperature. After a PBS rinse, they were incubated in a goat-anti-mouse IgG conjugated to AF488 (1:100, Jackson ImmunoResearch; 115-545-146) for 1 h at room temperature. After a PBS rinse, the sections were again blocked in 20% normal goat serum, followed by incubation with a mouse antibody to dystrophin (1:200, Sigma, St. Louis, MO; D8043). The sections were rinsed in PBS, blocked in 20% normal serum, and incubated in goat- anti-mouse IgG conjugated to Cy3 (1:500; Jackson ImmunoResearch; 115-166-006). The analysis consisted of determining the number of Pitx2-positive nuclei, defined as myonuclei if they were within the dystrophin ring marking the sarcolemma or as myogenic precursor cells if they were located outside the dystrophin ring. This was performed at two locations between the tendon-end and the midbelly region, and the results were averaged for each muscle analyzed, and these averaged counts were used to determine statistical significance.

Quantification was performed using the Bioquant Life Science Image Analysis System (Bioquant Image Analysis Corporation). For all analyses, a minimum of three slides were analyzed for each set of horizontal extraocular muscles. Each set was averaged, and these were used to determine the overall average for location and treatment for the GDNF-treated medial rectus muscles, the lateral rectus muscles on the same eye, as well as the contralateral medial and lateral rectus muscles.

All morphometric analyses were performed masked to the muscle being analyzed. Data are reported as mean ± standard error of the mean. Statistical analyses were performed using the Graphpad statistical program. For all data, ANOVAs were performed, followed by a Tukey’s post hoc multiple comparison test. Significant difference was defined as p ≤ 0.05.

## Supplementary information

Supplementary Video.
